# A novel helper-dependent adenovirus-based cell culture model for Hepatitis C virus replication and production

**DOI:** 10.1186/1743-422X-10-273

**Published:** 2013-08-30

**Authors:** Xiaojun Zhou, Yang Zeng, Junfeng Li, Yan Guo, Yuanhui Fu, Jinsheng He, Shihui Sun, Yusen Zhou

**Affiliations:** 1 State Key Laboratory of Pathogen and Biosecurity, Beijing Institute of Microbiology and Epidemiology, Beijing, China; 2 Laboratory Animal Center of the Academy of Military Medical Science, Beijing, China; 3 College of Life Sciences & Bioengineering, Beijing Jiaotong University, Beijing, China

**Keywords:** Hepatitis C virus, Cell-culture model, Helper-dependent adenovirus

## Abstract

**Background:**

By using the hepatitis C virus (HCV) genotype 2a JFH-1 or its chimeric strains, a HCV infection system has been previously developed through several methods– such as in vitro-transcribed JFH1-RNA transfection or stable transfection of the JFH1 cDNA into human hepatoma Huh-7 cell line or its derivatives. However, other reliable methods for delivery of the HCV genome into cells are still worth trying. The helper-dependent adenovirus (HDAd) is devoid of all viral coding sequences and has a package capacity of 37 kb, which is suitably large for the delivery of the HCV genome. Here we report a new method for delivery of the HCV genome into Huh-7 and HepG2 cells by using the HDAd vector.

**Results:**

Our results demonstrated that the infection of Huh-7 cells with the HDAdJFH1 virus led to efficient HCV replication and virion production. We found that the HCV viral RNA levels could reach 107 copies per milliliter (ml) in the culture medium. HDAdJFH1-infected Huh-7 cells could be cultured for 8 passages with the culture medium remaining infectious for naïve Huh-7 cells throughout this period. This infection system proved effective for evaluating the anti-HCV effects of IFN-α in Huh-7 cells. Co-infection of HepG2 cells with the HDAdJFH1 and HDAdmiR-122 virus also resulted in HCV expression and replication.

**Conclusion:**

This is the first report of an HDAd-based strategy for HCV replication and production in vitro.

## Background

Hepatitis C virus (HCV) is a 9.6 kb positive-strand RNA virus and a member of the flavivirus family [1]. It is estimated that approximately 170 million people worldwide are persistently infected by HCV, which can result in hepatic fibrosis, cirrhosis and hepatocellular carcinoma [2]. Current interferon-α based therapy, in combination with ribavirin, has limited efficacy in only approximately 50% of patients and has severe side effects [3]. HCV replication takes place in the cytoplasm, and the 9.6 kb RNA genome encodes a polyprotein localized to the rough endoplasmic reticulum (ER), where it is cleaved into at least 10 structural (C, E1, E2, and P7) and nonstructural (NS2, NS3, NS4A, NS4B, NS5A, and NS5B) proteins that play important roles in virus replication, assembly and pathogenesis [4].

HCV research has been hampered by the lack of adequate *in vitro* and *in vivo* model systems. Replicons have been utilized for studying HCV RNA replication, but these are not useful for studying aspects of virion production and infection [5]. In 2005, researchers discovered a genotype 2a isolate JFH1 from a Japanese patient with fulminant hepatitis that could exhibit complete the virus life cycle after transfection of *in vitro* transcribed full-length JFH1 RNA into Huh-7 or Huh-7.5 cells. This system is also able to produce infectious viral particles in cell culture (HCVcc) [6,7]. Furthermore, it was found that stable human hepatoma cell lines containing a chromosomally integrated cDNA copy of the JFH1 genome with a hepatitis delta virus ribozyme at the 3′ end can constitutively produce infectious viral particles [8]. These methods have proven to be effective in generating infectious HCV cell culture models in Huh-7 cell line and its derivatives.

In contrast to Huh-7 cells, the hepatocellular carcinoma derived HepG2 cells polarize and would thus permit the investigation of how cell polarization impacts the HCV life cycle [9]. However, HepG2 cells does not express endogenous miR-122, a liver-expressed miRNA which is required to support HCV RNA replication [10], and weakly supports HCV replication [11]. Although a recent study has indicated that HepG2 cells expressing miR-122 can support the entire HCV life cycle [11], the efficiency of HCV replication and virion production still needs increasing. Thus other potential methods besides transfection for delivery of the HCV genome into cells are still worth trying especially when the cells, such as HepG2 cells, possess a relatively lower transfection efficiency.

Adenoviruses (Ads) are non-enveloped double-stranded DNA viruses, which can mediate efficient transduction and expression of foreign genes in cells [12]. The helper-dependent adenovirus (HDAd) possesses the same ability to deliver foreign DNA into cells as earlier generation adenoviruses (Ads); furthermore, HDAd vectors are devoid of all viral coding sequences and have cloning capacities of up to 37 kb [13], which makes it possible to introduce large genes into cells using HDAd vectors. Lacking all viral coding sequences, it displays only minimal immunogenicity and negligible side-effects and allows for long-term transgene expression in animal models for delivery of transgenes into the liver, skeletal muscle, myocardium or brain [14]. Furthermore, it does not integrate into the host genome, which makes them a promising class of potential delivery vehicles for human gene therapy [15].

In this study, we developed a HDAd vector containing the full-length JFH1 genome and an HDV ribozyme sequence located at the 3′ end of the JFH1 genome. Our results demonstrate that the HDAd vector was able to efficiently deliver the HCV genome into Huh-7 cells and HepG2 cells, in which infectious HCV particles could be produced *in vitro*. To our knowledge, an efficient HDAd-based method for introducing the full-length 9.6 kb HCV RNA genome into hepatocytes has not been previously described. Therefore, this is the first report of an *in vitro* HDAd-mediated HCV genomic replication and production system.

## Results

### Construction of a helper-dependent adenoviral vector expressing the HCV RNA genome

**Figure 1:**
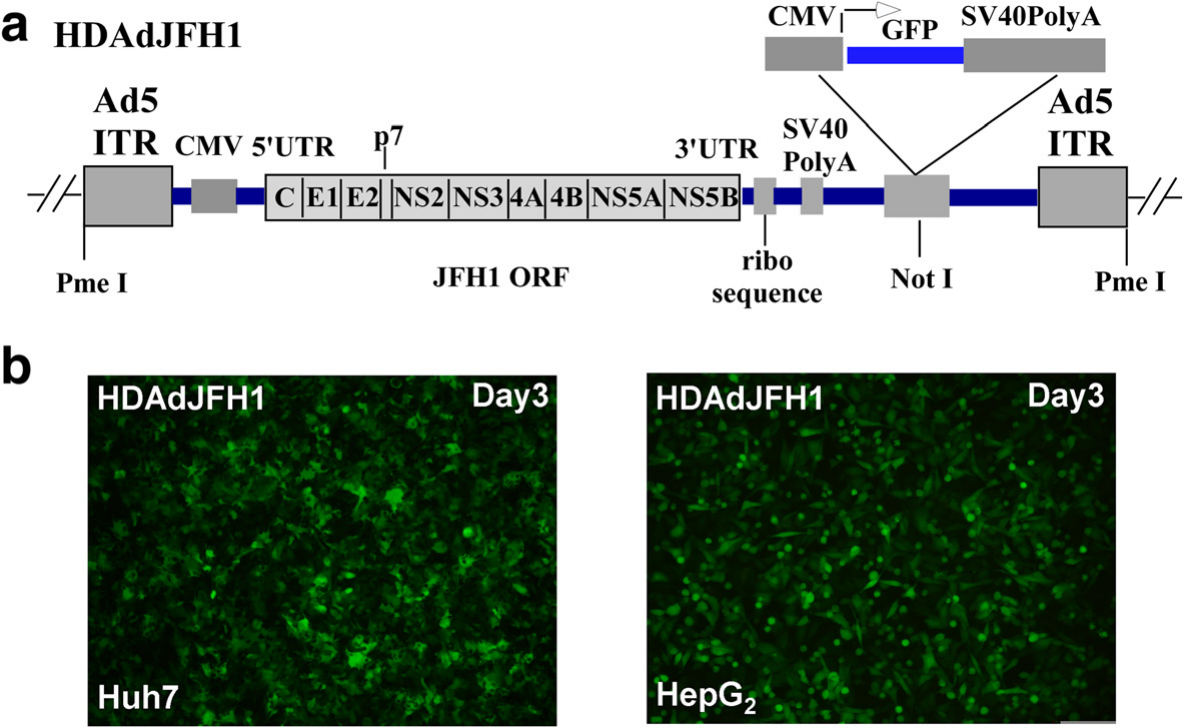
**Infection of Huh-7 cells and HepG2 cells by the HDAdJFH1 virus. (a)**. A diagram depicting the genetic makeup of HDAdJFH1. The transcriptional expression of the JFH1 RNA genome is under the control of the CMV promoter and the HDV ribozyme. The GFP expression cassette is under the control of a second CMV promoter and contains an SV40 polyA tail. **(b)**. GFP expression in HDAdJFH1-infected Huh-7 cells and HepG2 cells on day 3 p.i. Magnification, ×10 **(b)**.

An Ad5 vector has been previously used for the introduction of the hepatitis B viral genome into cultured cells and mice, and it was found that high-titer hepatitis B virions were secreted into the culture medium of infected hepatoma cells and the sera of infected mice [16]. However, the size of the transgene that can be delivered by the conventional Ad5 vector is limited to 8.1-8.2 kb [17], which is nearly the size of the HCV replicon. Although an Ad5/35 chimera vector was recently used to generate a HCV subgenomic-replicon construct [18], there have been no reports of the packaging and transfer of the full 9.6 kb HCV genome *in vitro* or *in vivo*. In this study, we aimed to develop a simple and reliable method for packaging and delivering the HCV genome into cells to establish a novel HCV infection system. To this end, we chose to utilize the genotype 2a JFH1 HCV clone due to its efficient replication in human hepatoma [19] and mouse cells [20]. The full-length JFH1 RNA genome is under the control of a 5′ minimal CMV promoter in the HDAd vector, and the 3′ terminus of the transcript is processed by an HDV ribozyme sequence. The helper-dependent adenovirus plasmid also contained a GFP expression cassette (Figure 1a).

### Amplification of the HDAdHCV virus and infection of cells

As quantified by absorbance, the titer of the amplified and purified HDAdJFH1, HDAdJFH1/GND, HDAdmiR-122 and HDAdGFP virus were found to be 2×10^11^, 3×10^11^, 1×10^11^, 2×10^11^viral particles/ml, respectively. Huh-7 cells and HepG2 cells were infected by the HDAds at a ratio of 200 viral particles/cell. GFP expression was observed in the infected Huh-7 and HepG2 cells at 3 days p.i. (Figure 1b). At 3 days p.i, nearly all the Huh-7 and HepG2 cells were infected with the HDAdJFH1 virus and had high levels of GFP expression.

### Detection of HDAd-mediated HCV protein expression and RNA replication

**Figure 2:**
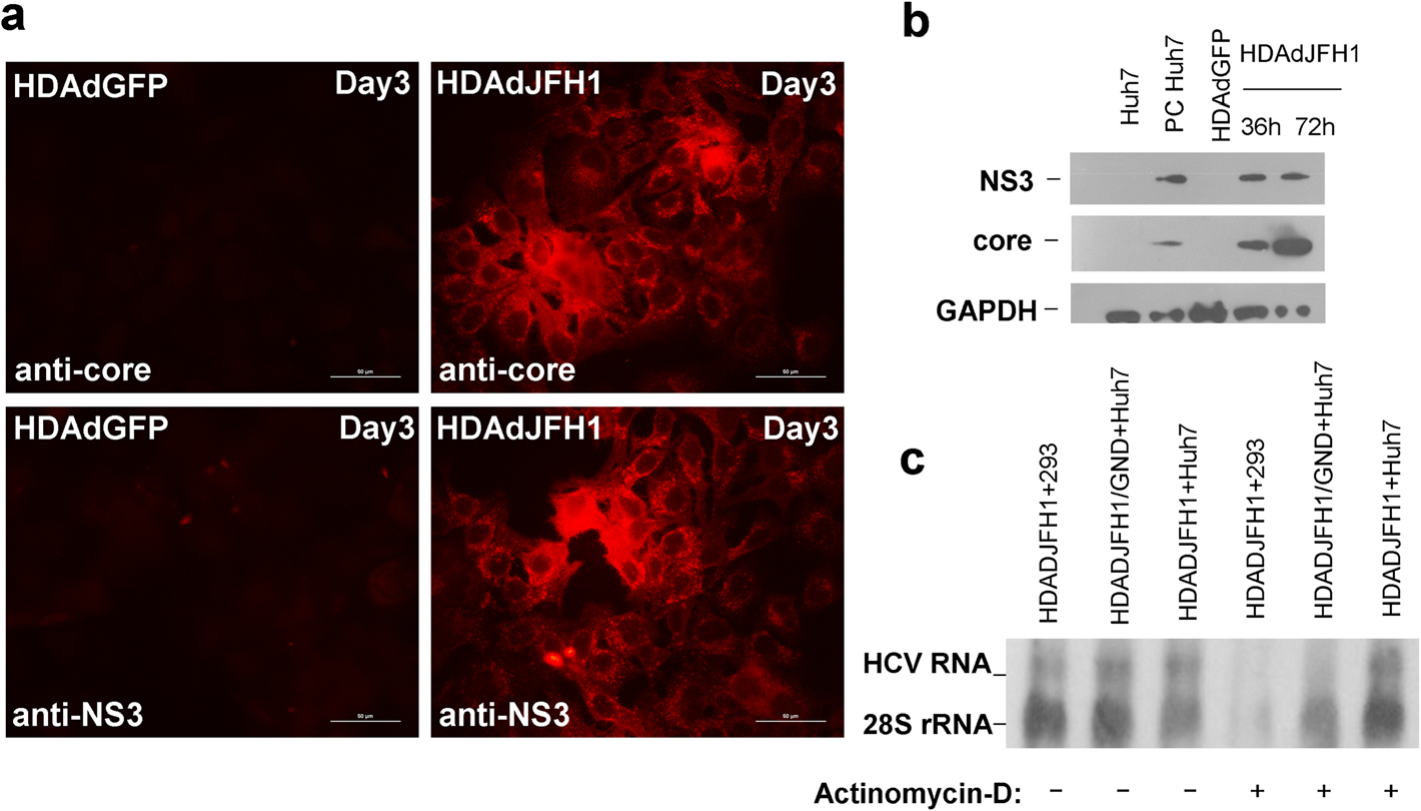
**HCV expression and replication in HDAdJFH1-infected Huh-7 cells. (a)**. Immunofluorescence assays (IFAs) of the HCV core and NS3 proteins in Huh-7 cells on day 3 p.i. HDAdGFP-infected Huh-7 cells served as a negative control. **(b)**. Western blots detecting the HCV core and NS3 proteins in HDAdJFH1-infected Huh-7 cells at 36 h and 72 h p.i. Huh-7 cells transfected with *in vitro*-transcribed JFH1 RNA served as a positive control. HDAdGFP-infected Huh-7 cells served as a negative control. **(c)**. Northern blots detecting HCV RNA and 28S rRNA in HDAdJFH1-infected Huh-7 cells, HDAdJFH1/GND-infected Huh-7 cells and the HDAdJFH1-infected 293 cells in the presence of actinomycin-D. A group of infected cells without actinomycin-D served as controls. Magnification, ×40 **(a)**.

HDAd can mediate the efficient delivery and strong expression of foreign DNA sequences *in vitro*[21]. Our results indicate that HDAd can also mediate the efficient expression of HCV proteins in HDAdJFH1-infected Huh-7 cells. The expression of HCV core and NS3 proteins was confirmed by immunofluorescence staining and western blotting using core- and NS3-specific monoclonal antibodies. At 3 days p.i., immunofluorescence staining for HCV core and NS3 were both positive (Figure 2a). High levels of HCV core and NS3 proteins were also detected by western blotting at 36 h and 72 h p.i., respectively (Figure 2b). The expression of HCV NS3 protein was still detectable at 20 days p.i. (data not shown), demonstrating the long-term expression of the HCV genome.

**Figure 3:**
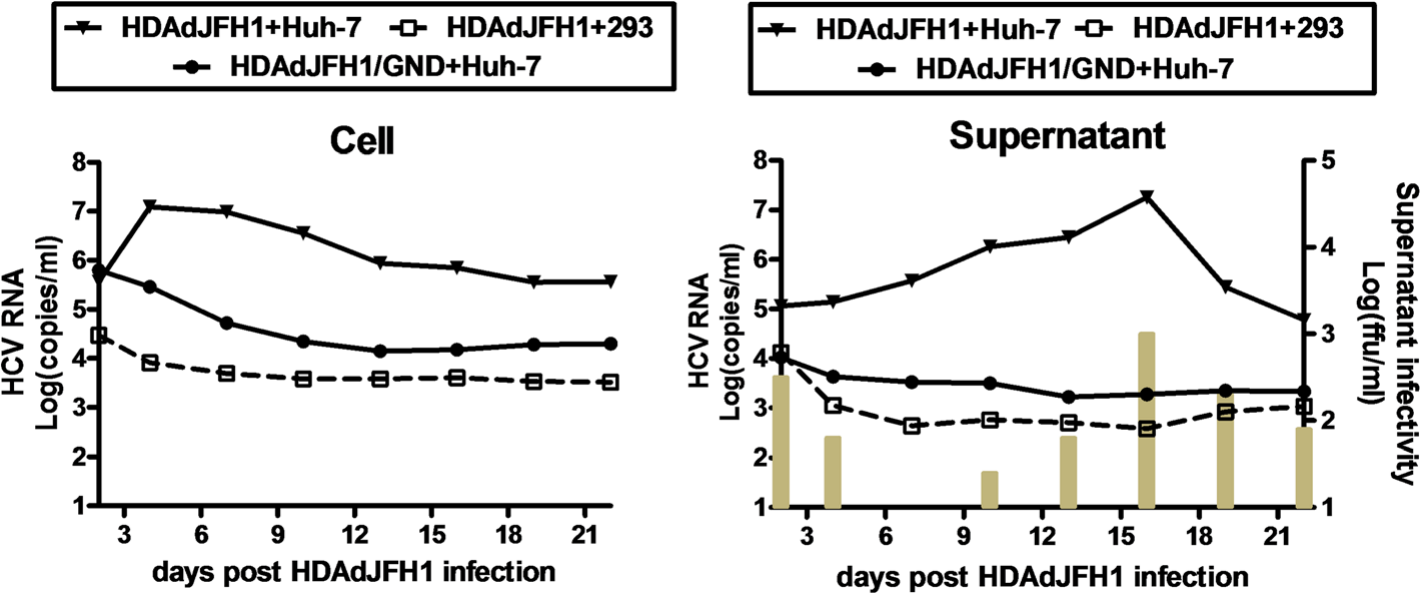
**Quantification of the HCV RNA levels in the cells and supernatants.** HCV RNA levels were analyzed by RT-qPCR with the HDAdJFH1/GND-infected Huh-7 cells and the HDAdJFH1-infected 293 cells as controls. The supernatant infectivity titers were determined in naïve Huh-7 cells and were expressed as ffu/ml (indicated with bars).

Actinomycin-D has been shown to have the ability to inhibit transcription [22]. Thus, actinomycin-D was added in the cell culture medium and northern blot was performed to confirm replication of HCV genome. The concentration of actinomycin-D was maintained 5 μg/ml in the medium for 16 hours in HDAdJFH1, HDAdJFH1/GND-infected Huh-7cells and HDAdJFH1-infected 293 cells since 6 hours p.i. After actinomycin-D treatment, expression of intracellular HCV RNA can be detected in the HDAdJFH1-infected Huh-7 cells, compared with those in the HDAdJFH1/GND-infected Huh-7 cells and HDAdJFH1-infected 293 cells (Figure 2c). The results indicated that actinomycin-D inhibited the HDAd-mediated HCV genome transcription in HDAdJFH1/GND-infected Huh-7 cells and HCV replicated in the HDAdJFH1-infected Huh-7 cells. Quantitative RT-PCR analysis also showed an increase of HCV RNA levels in the cell culture medium since HDAdJFH1 infection of Huh-7 cells (Figure 3). Together, these results demonstrated the replication of HCV genome in the HDAd-JFH1-infected Huh-7 cells.

### Determination of the infectivity of HDAdJFH1-infected Huh-7 cell culture medium

**Figure 4:**
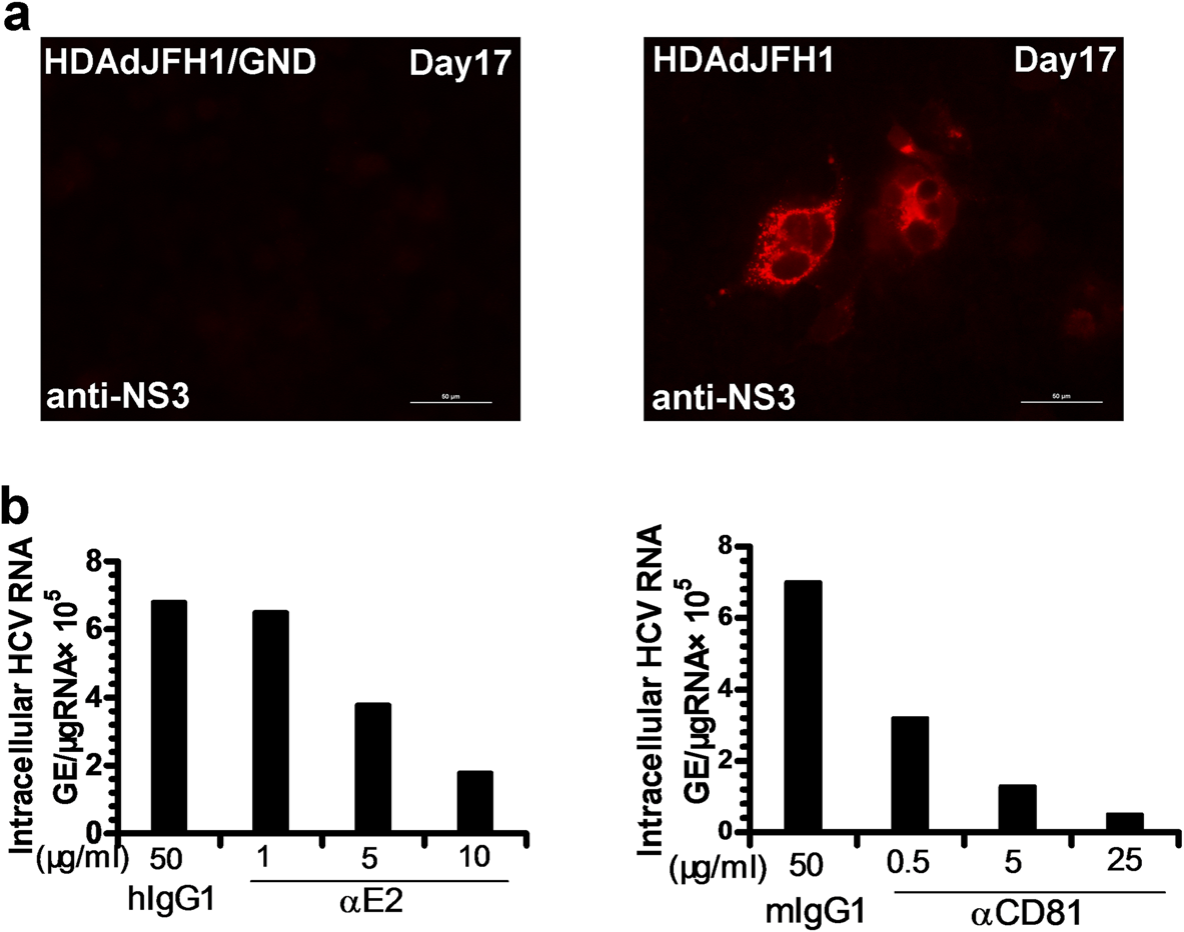
**Infectious HCV production in Huh-7 cells following HDAdJFH1 infection. (a)**. IFA of the HCV NS3 protein in Huh-7 cells infected with supernatant from HDAdJFH1-infected Huh-7 cells that were collected on day 17 p.i. Huh-7 cells infected with supernatant from HDAdJFH1/GND-infected Huh-7 cells collected on day 17 p.i. served as negative controls. **(b)**. Inhibition of HCV infection by the anti-E2 and anti-CD81 antibody. The infectious HCV supernatants were pre-incubated with the indicated concentrations of anti-E2 or control mouse IgG antibodies for 1 h at 37°C before addition to naïve Huh-7 cells. Huh-7 cells were pre-incubated with the indicated concentrations of anti-human CD81 or control mouse IgG antibodies for 1 h at 37°C before addition of the infectious HCV supernatant. Total cellular RNA was analyzed by RT-qPCR on day 3 p.i.

We found that HCV RNA levels in the cell culture medium of the HDAdJFH1-infected Huh-7 cells first increased to a maximal level at 16 days p.i. and then decreased in the following days. While HCV RNA levels in the supernatant of the HDAdJFH1/GND-infected Huh-7 cells decreased gradually (Figure 3). Based on these findings, we wondered whether HDAd-mediated HCV virions were present in the cell culture medium. To determine the infectivity of the medium from the passaged HDAdJFH1-infected Huh-7 cells, we infected naïve Huh-7 cells with the medium for 1 h at 37°C; we subsequently determined the expression levels of the HCV NS3 protein using IFA. As shown in Figure 4a, naïve Huh-7 cells were infected with medium collected at day 17 p.i., cultured for another 3 days and then assayed for HCV NS3 protein expression using IFA. Naïve Huh-7 cells served as a negative control. Huh-7 cells were also infected with medium from HDAdJFH1/GND-infected Huh-7 cells to confirm that HDAdJFH1 infection mediated HCV replication and infectious HCV secretion into the medium of HDAdJFH1-infected Huh-7 cells.

To determine in more detail the infectious titer of HCV, the medium was serially diluted in 10-fold increments and used to infect Huh-7 cells. The infectious titer was determined by counting the number of cell foci that stained positive for HCV NS3 protein at the lowest dilution point, which was then multiplied by the dilution factor (n-fold) [6]. The infectious HCV titer of the medium reached a maximal level of 1×10^3^ ffu/ml at 16 days p.i. (Figure 3) and was comparable to the infectious HCV titer in Huh-7 cell by conventional transfection of *in vitro* transcribed JFH1 RNA, which normally only reaches 10^2^~10^3^ ffu/ml.

### HCV infection is inhibited by anti-E2 and anti-CD81 antibodies

Previous studies using pseudotyped viruses expressing HCV E1/E2 have suggested that the interaction between E2 and CD81 is crucial for viral entry [23,24]. To further investigate the infectivity of the HCV virions, we performed infectivity-neutralization and inhibition experiments using monoclonal antibodies specific for HCV E2 and CD81. Prior to addition to the Huh-7 cells, the infectious HCV supernatant was pre-incubated with a dilution series of a mouse monoclonal antibody specific for HCV E2. An irrelevant human IgG1 antibody served as a negative control. In another experiment, the Huh-7 cells were pre-incubated with CD81 antibodies before the infectious HCV supernatant was added. The irrelevant mouse IgG1 antibody served as a negative control. For both the E2 and CD81 antibody treatments, the intracellular HCV RNA levels were observed to decrease in a dose-dependent manner by 3 days p.i. A concentration of 5 μg/ml of anti-E2 antibody was sufficient to cause a 50% reduction in the intracellular HCV RNA level by 3 days p.i., whereas only 0.5 μg/ml of anti-CD81 antibody was sufficient to cause the same reduction (Figure 4b). These results are consistent with the previously reported findings [6,7] and confirm the infectivity of the cell culture medium.

**Figure 5:**
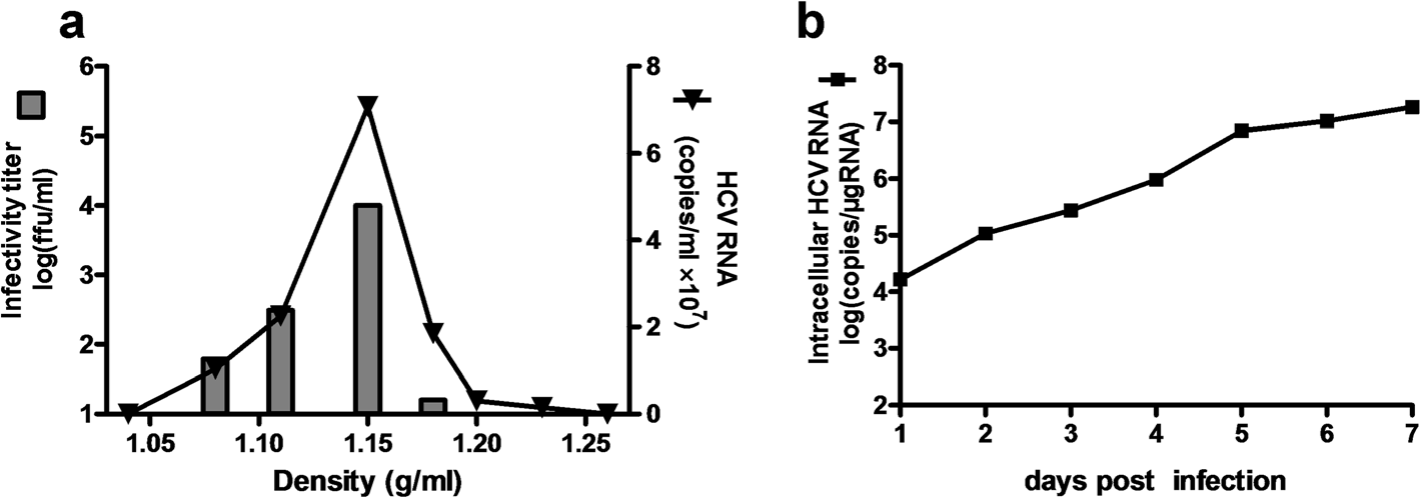
**HCV infectivity determination by density gradient analysis and infection kinetics assay. (a)**. Density gradient analysis of produced HCV particles. Concentrated culture medium collected from HDAdJFH1-infected Huh-7 cells was fractionated, HCV RNA levels and HCV infectivity titers in each fraction was determined. **(b)**. Infection of naïve Huh-7 cell by the supernatant from the HDAd-JFH1-infected Huh-7 cells and infection kinetics assay analysis.

To characterize the produced HCV virions, the culture medium of the HDAdJFH1-infected Huh-7 cells was subjected to sucrose density gradient centrifugation. The fractions were analyzed for HCV RNA levels, as well as the infectivity titers. As shown in Figure 5a, a peak for HCV RNA level and infectivity titer was found in fraction 4, which has a density of 1.15 g/ml. This density is almost consistent with the published density of HCV virions [8,25]. The infection kinetics assay showed that the cellular HCV RNA levels increased to 10^7^ copies within 7 days p.i. (Figure 5b). These results further confirm the production of infectious HCV virions in this HDAd-mediated system.

### Inhibition of HCV replication by IFN-α

**Figure 6:**
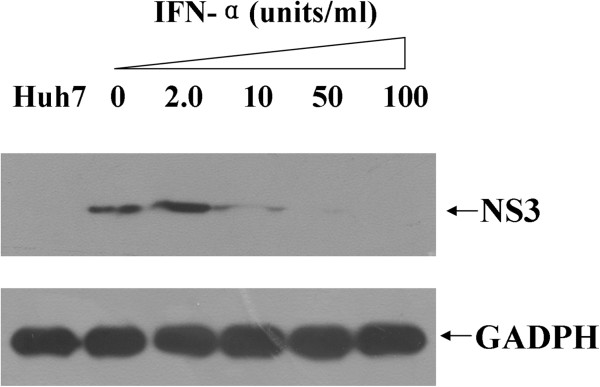
**IFN-α inhibition of HCV replication in HDAdJFH1-infected Huh-7 cells.** The effects of IFN-α on HCV replication were determined by western blot analysis of HCV NS3 expression.

Previous studies have shown that IFN-α can successfully inhibit HCV replication both in Huh-7 cells transfected with JFH1 RNA [6] and in Huh-7 cells with a stably integrated HCV genome [8]. Therefore, we investigated the inhibitory effects of IFN-α on HCV replication in our HDAd-mediated infection system. Beginning at 8 days p.i., HDAdJFH1-infected Huh-7 cells were incubated with DMEM containing different concentrations of IFN-α for 3 days, and levels of the NS3 protein were determined by western blotting. IFN-α efficiently inhibited HCV replication in HDAdJFH1-infected Huh-7 cells (Figure 6). Therefore, it is likely that this HDAd-mediated HCV infection system is suitable for the evaluation of new anti-HCV drugs and therapeutic strategies.

### HDAdJFH1 and HDAdmiR-122 virus co-infection mediate HCV expression and replication in HepG2 cells

**Figure 7:**
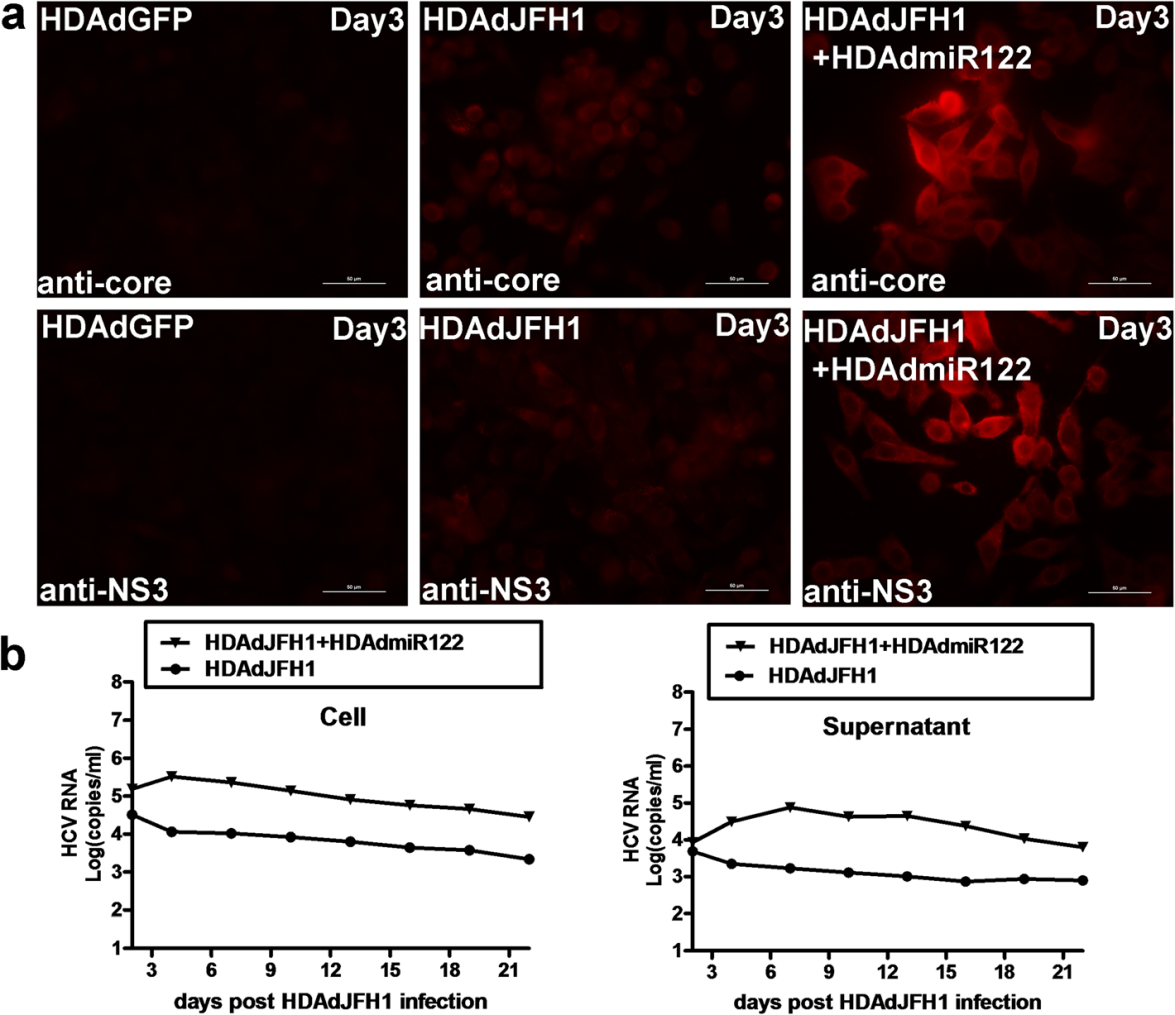
**HCV expression and replication in HepG2 cells co-infected with HDAdJFH1 and HDAdmiR122 virus. (a)**. IFAs of the HCV core and NS3 proteins in HepG2 cells co-infected with HDAdJFH1 and HDAdmiR122 virus on day 3 p.i. HDAdGFP-infected HepG2 cells and HDAdJFH1–infected HepG2 cells served as controls. **(b)**. Quantification of the HCV RNA levels in the HDAdJFH1 and HDAdJFH1/GND-infected HepG2 cells and supernatants. Magnification, ×40 **(a)**.

HepG2 cells does not express endogenous miR-122, a liver-expressed miRNA which is required to support HCV RNA replication [10], and weakly supports HCV replication [11]. A recent study has indicated that HepG2 cells expressing miR-122 can support the entire HCV life cycle [11]. In this study, we tested whether the HDAd system can work in HepG2 cells. Results showed that co-infection of HepG2 cells with HDAdJFH1 and HDAdmiR-122 virus enhances HCV core and NS3 protein expression (Figure 7a). Kinetic analysis of HCV RNA levels in the co-infected HepG2 cells and culture medium were performed. Compared with the HDAdJFH1/GND infection control, HDAdJFH1 and HDAdmiR-122 co-infection resulted in relatively weak replication of HCV genome, as shown in Figure 7b.

## Discussion

Hepatitis C virus (HCV) is a positive-strand RNA virus that was first identified in 1989. Since then, both basic and pharmaceutical research on HCV has been hampered by a lack of viral culturing systems [4]. Early studies on HCV mainly focused on the structure and function of the individual viral genes, and later studies utilized HCV subgenomic replicons, which were more effective for HCV RNA replication research and antiviral development [20,26]. However, the replicon system is not suitable for HCV life-cycle research due to this system’s inability to produce infectious HCV virions. In 2005, it was reported that RNA generated from the JFH1 viral genome can produce infectious HCV virions when transfected into Huh-7 cells [7]. Both transient and stable transfections with DNA vectors expressing the full-length HCV genome have also been investigated [8,25] and in these studies it was found that the HDV ribozyme plays an important role in cleavage of the HCV RNA product.

It appears that efficient delivery of the HCV RNA genome, for example by electroporation or stable transfection, is vital for the efficient replication and production of the HCV virus. Here, we report on the use of the helper-dependent adenoviral (HDAd) vector, which can mediate high-efficiency transduction of one or more foreign genes into cells [27]. Adenoviruses (Ads) are non-enveloped double-stranded DNA viruses, replication and assembly of which take place in the nucleus. Adenovirus vectors are most effective at mediating the transfer of foreign DNA into immortalized and primary cells. In the liver, they predominantly infect hepatocytes [21], where hepatitis-virus replication and infection take place. Adenoviral vectors have been used for the transfer of the genome of the hepatitis B virus into cultured cells and mice [16]. However, the 9.6 kb HCV RNA genome was too large to be introduced into early generation Ad vectors. HDAds are constructed by removing all viral sequences from earlier generation Ad vectors except for the packaging sequences and the inverted terminal repeats, which eliminates viral gene expression, liver toxicity, and the cellular immune responses elicited by Ad vectors [28]. As most of the viral coding sequences are deleted, HDAds have a large delivery capacity of up to 37 kb [29]. As a comparison, the upper size limit of transgenes that can be delivered with the conventional Ad5 vector is 8.1-8.2 kb [17] – approximately the size of the HCV replicon. Thus, it has been difficult to package the HCV replicon into Ad5, although an Ad5/35 chimera vector has recently been used to generate an HCV subgenomic-replicon construct [11]. To our knowledge, a convenient and efficient method for transferring the 9.6 kb HCV RNA genome into cells by HDAds has not been previously reported.

In this study, we developed an HDAd vector containing the full-length JFH1 genome and an HDV ribozyme sequence. HDAdJFH1 virus with a high infectious titer was prepared and used to infect Huh-7 cells *in vitro*, and we observed both long-term expression and replication of HCV genome (Figures 2 and 3). HCV replication was confirmed by de novo RNA synthesis in the presence of actinomycin-D. The HCV RNA levels in the supernatants increased to a maximal level of about 10^7^ copies/ml at 16 days p.i. when many cells became rounded and floated in the culture medium, a similar cytopathic effect that can be observed on the JFH1-transfected Huh-7 cells [30]. Ten days after infection, the supernatant from HDAdJFH1-infected Huh-7 cells was found to be infectious and the infectivity of HCV could be inhibited by anti-E2 and anti-CD81 antibodies in a dose-dependent manner (Figure 4b). As the interaction between E2 and CD81 is vital for HCV entry [31,32], we believed that HCV particles were being secreted into the supernatant of HDAdJFH1-infected Huh-7 cells. Sucrose density gradient analysis showed that the virus produced owns a density which is similar to published data for HCV virions [25]. Furthermore, we found that IFN-α efficiently inhibited HCV replication (Figure 6), further validating the potential of this system for the evaluation of HCV antiviral drugs. Taken together, our findings demonstrate long-term replication of the HCV genome and production of infectious HCV virions in this helper-dependent adenovirus-mediated system in Huh-7 cells.

MicroRNAs (miRNA) are 21~22 nucleotide RNA molecules that are expressed in a wide range of eukaryotic organisms that are predicted to downregulate the expression of the endogenous target genes by reducing mRNA stability or translation [33]. MiR-122 is liver-specific and required for efficient HCV RNA replication [10,34]. HepG2 cells does not express endogenous miR-122 and weakly supports HCV replication [11]. A recent study has indicated that HepG2 cells expressing miR-122 can support the entire HCV life cycle [11]. In this study, our results also showed that HDAd-mediated miR-122 expression was necessary for the HDAd-mediated efficient HCV expression and replication (Figure 7).

Although the method based on *in vitro*-transcribed JFH1-RNA transfection is very well established so far, it is less efficient for primary hepatocytes which are difficult to transfect. Helper-dependent adenovirus can infect a variety of different cell types, including hepatic cell lines, primary hepatocytes [35], as well as quiescent, non-dividing, or terminally differentiated somatic cells [36], with high expression efficiency. Thus, by using this HDAd system, HCV replication or infection can be studied in the above cell types *in vitro*. So far, HCV can only infect human and chimpanzees *in vivo* and the host range for HCV infection is narrow. Although human liver chimeric mice can be infected by HCV, it is immunodeficient with a high mortality rate and the source of human primary hepatocytes is limited. And it is important to generate a novel immunocompetent mouse model which can support HCV replication and infection. Although expression of the four humanized HCV receptors in mice enhance its susceptibility to HCV infection [37,38], it is still not successful for mediating HCV infection in mice. Several studies have shown that HDAds are able to mediate high-level and long-term transgene expression in animal models, especially for the liver-targeted gene therapy [13,14]. So it is probable that HDAd can mediate efficient delivery of the HCV genome into the mouse livers, but whether HDAd can mediate HCV genomic replication or even virion production in mice is worth being investigated. Also, since extrahepatic manifestations caused by hepatitis C virus infection exists clinically [39], animal model for studying HCV extrahepatic manifectations still lacks. And this HDAd-mediated HCV genomic replication system provides a possible strategy for generating an HCV extrahepatic replication model *in vivo*.

## Conclusion

This is the first report of an *in vitro* HDAd-mediated HCV genomic replication and production system, which would allow a broader examination of interactions between HCV and host cell biology, as well as provide tools for anti-HCV drug evaluation.

## Methods

### Helper-dependent adenoviral vector construction

The plasmid pJFH1 contains the full-length JFH1 cDNA downstream of the T7 RNA promoter [7]. To replace the T7 promoter from pJFH1 with CMV promoter, a PCR fragment, amplified 5′ partial sequence of the HCV genome in pJFH1 using the chimeric forward primer 5′-ccgaattcgagctcggtacccgg-3′ which consists of two segments lying beside the upstream and downstream of T7 promoter and reverse primer 5′-caccggttccgcagaccac-3′, was inserted into pJFH1 between the restriction enzyme sites for *EcoR* I and *Age* I that locates upstream and downstream of the T7 promoter respectively. The resulting construct was designated pJFH1(ΔT7). A hepatitis delta virus (HDV) ribozyme DNA sequence was synthesized and placed at the immediate 3′ end of pJFH1(ΔT7), to get pJFH1-ribo(ΔT7). The JFH1-ribo sequence was then inserted between the CMV promoter and SV40PolyA of the pSC11 plasmid by the restriction enzyme sites for *EcoR* I and *Hind* III. And this expression cassette was finally inserted into the HDAd vector between the restriction enzyme sites for *I-Ceu* I and *I-Sce* I. The resulting construct, HDAdJFH1, also contains a cassette for the expression of green fluorescent protein (GFP) [40]. HDAdJFH1/GND was constructed with the same strategy. And an empty HDAd plasmid expressing GFP (HDAdGFP) served as a control.

Using the Huh-7 cells derived total RNA-reverse transcribed cDNA as a template, the human genomic miR-122 precursor sequence was amplified by RT-PCR using the forward and reverse primers 5′-ccggaattcttcgtggctacagagttt-3′ and 5′-cccaagctttttatcgagggaaggatt-3′, and then inserted into the pSC11 plasmid by the restriction enzyme sites for *EcoR* I and *Hind* III. And this expression cassette was finally inserted into the HDAd vector between the restriction enzyme sites for *I-Ceu* I and *I-Sce* I. The resulting construct was designated HDAdmiR-122.

### Cells and cell culture methods used in this study

A modified version of the 293 cell line that expresses high levels of the Cre enzyme – known as the 116 cell line (kindly provided by Dr. Li-min Liu) – was maintained in MEM supplemented with 10% fetal bovine serum (FBS), 0.1 mg/ml hygromycin, 100 U/ml penicillin, 100 μg/ml streptomycin, and 2 mM L-glutamine (Invitrogen) [41]. The hepatoma cell lines Huh-7, HepG2, as well as non-hepatic 293 cell line, were obtained from ATCC (Manassas, VA) and maintained in DMEM supplemented with 100 U/ml penicillin, 100 μg/ml streptomycin, nonessential amino acids, and 10% FBS (Invitrogen).

### Amplification of the HDAdHCV virus and infection of cells

The helper-dependent adenoviral plasmids, including HDAdJFH1, HDAdJFH1/GND, HDAdmiR-122, HDAdGFP, were digested with the restriction enzyme *Pme*I to release both inverted terminal repeats and were then transfected into 116 cells with the calcium phosphate method, respectively [42]. Helper-dependent adenoviral particles were prepared by repeated amplification cycles in 116 cells with the helper virus AdNG163 (a kind gift of Dr. Li-min Liu; see above). After 4-6 rounds of amplification in 150-mm dishes, 116 cells suspended in a 3 L spinner flask were infected for large-scale preparation of HDAdJFH1, HDAdJFH1/GND, HDAdmiR-122, HDAdGFP. The viral particles were isolated by centrifugation in a cesium chloride gradient, followed by dialysis with three exchanges of 10 mM Tris-HCl (pH 8.0) at 4°C [43]. Virus particles were quantified by measuring the absorbance at 260 nm [44]. Huh-7, HepG_2_ and 293 cells were infected with the purified HDAds at 80% confluency with an MOI of 200 viral particles/cell and were passaged every 2 or 3 days.

### Western blot analysis

The HDAdJFH1-infected Huh-7 cells were lysed in protein sample buffer containing 50 mM Tris-HCl, 150 mM NaCl, 5 mM EDTA, 0.2 mM sodium orthovanadate, 1% Triton X-100, 1% sodium deoxycholate, and 1% sodium dodecyl sulfate; the buffer was also supplemented with aprotinin (2 μg/ml), pepstatin A (0.7 μg/ml), leupeptin (0.5 μg/ml), and PMSF (1 mM). For each sample, 30 μg of total protein was electrophoresed through a 10% sodium dodecyl sulfate-polyacrylamide gel and transferred onto a nitrocellulose membrane. The membrane was blocked by pre-incubation with 5% skim milk. The levels of the HCV core and NS3 proteins were determined using monoclonal antibodies specific to the core and NS3 proteins (Thermo Scientific), which were detected with a horseradish peroxidase-conjugated goat anti-mouse immunoglobulin G antibody (IgG, Pierce) and visualized with a chemiluminescent substrate (Pierce). The level of the GAPDH protein served as an internal control, and this protein was detected with an anti-GAPDH monoclonal antibody (Sigma).

### Immunofluorescence assays (IFAs)

The HDAdJFH1-infected Huh-7 and HepG2 cells were grown overnight on coverslips in a 6-well culture plate. Cells were washed with 1× phosphate-buffered saline (PBS), fixed with chilled acetone, and blocked with 1% bovine serum albumin and 1% goat serum in PBS. The localizations of the HCV core and NS3 proteins were visualized in the fixed cells by incubation with core- and NS3-specific monoclonal antibodies and a secondary goat anti-mouse IgG antibody conjugated to DyLight™594 fluorescein (used at a 1:800 dilution) (Beijing Golden Bridge Biotechnology, Beijing, China). The HDAdGFP-infected Huh-7 and HepG2 cells served as a negative control. The coverslips were then mounted onto slides, and the HCV proteins were visualized with a Zeiss Axioplan 2 fluorescence microscope.

### Quantification of HCV RNA levels

Total cellular RNA was extracted from infected cells using the RNeasy RNA isolation kit (Qiagen). Total RNA was extracted from the supernatants of the infected cells using the QIAamp Viral RNA Mini Kit (Qiagen). The RNA concentration was determined by spectrophotometry. RT-qPCR was performed as described elsewhere [6], and the HCV levels were determined relative to a standard curve created using serial dilutions of a plasmid containing the HCV JFH1 cDNA.

### Northern blot analysis of intracellular viral RNA levels

To confirm the replication of HCV genome, actinomycin-D (Sigma) was maintained in the cell culture medium at a concentration of 5 μg/ml for 16h in HDAdJFH1, HDAdJFH1/GND-infected Huh-7cells and HDAdJFH1-infected 293 cells since 6 hours p.i.. Total cellular RNA was extracted from infected Huh-7 cells using the RNeasy RNA isolation kit (Qiagen). Four micrograms of isolated RNA was separated using a 1% agarose gel containing formaldehyde, blotted onto a positively charged nylon membrane (Hybond-N+, GE), and immobilized with a Stratalinker UV crosslinker (Stratagene). The DNA probes complementary to a region of the HCV 5′UTR and human 28S rRNA was synthesized using the DIG High Prime DNA Labeling and Detection Starter Kit II (Roche).

### HCV infection and infectivity neutralization

Naïve Huh-7 cells in a 6-well culture plate were infected with 1 ml of cultured supernatant from HDAdJFH1-infected Huh-7 cells. At 3 h postinfection (p.i.), the supernatants were replaced with 2 ml of DMEM containing 10% FBS, and the cells were incubated at 37°C for 3 days prior to the protein and RNA analysis. At 3 days p.i., the HCV infectivity was determined by IFA for the HCV core and NS3 proteins using core- and NS3-specific monoclonal antibodies, respectively. Titration of infectious HCV was performed by focus forming assay. Cell supernatants were serially diluted 10-fold in complete DMEM and used to infect naïve Huh-7 cells. The level of infectious HCV titer was determined by the average number of NS3-positive foci detected at highest dilutions at 3 days p.i.. For the infectivity-neutralization experiments, monoclonal antibodies specific to HCV E2 and CD81 (Santa Cruz Biotechnology) were diluted with the HCV-containing culture medium at 3 days p.i.; normal mouse IgG1 (Santa Cruz Biotechnology) antibodies were used as negative controls. The effects of the E2 and CD81 monoclonal antibodies on HCV infectivity and replication were determined by RT-qPCR.

### Sucrose density gradient analysis

We collected the cell culture medium of the HDAdJFH1-infected Huh-7 cells at 16 days p.i. using low-speed centrifugation and passed it through a 0.45-μm filter. Then the filtrate was layered on a sucrose gradient (60%~10%, wt/vol) and centrifuged it for 16 h. The fractions were harvested and analyzed for the HCV RNA levels and infectivity titers.

### IFN-α inhibition of HCV replication in HDAdJFH1-infected Huh-7 cells

For the IFN-α inhibition experiments, Huh-7 cells were infected with HDAdJFH1 and incubated with DMEM containing increasing concentrations of IFN-α (Sigma) for 3 days. The effects of IFN-α on HCV replication were determined using western blot analysis of HCV NS3 expression.

## Abbreviations

HCV: Hepatitis C virus; cDNA: Complementary DNA; HCVcc: infectious HCV in cell culture; HDAd: Helper-dependent adenovirus; ml: milliliter; IFN-α: Interferon-α; RNA: Rinonucleic acid; ER: Endoplasmic reticulum; UTR: Untranslated region; ORF: Open reading frame; miRNA: microRNA; IFA: Immunofluorescence assay; RT-PCR: Reverse transcription-polymerase chain reaction; ffu: foci forming unit; p.i.: postinfection; DMEM: Dulbecco’s Modified Eagle Medium; CMV: Cytomegalovirus; FBS: Fetal bovine serum; ATCC: American type culture collection; MOI: Multiplicity of infection; EDTA: Ethylenediaminetetraacetic acid; PMSF: Phenylmethanesulfonyl fluoride; RT-qPCR: Real-time quantitative polymerase chain reaction; DIG: Digoxigenin.

## Competing interests

The authors have declared that no competing interests exists.

## Authors’ contributions

Conceived and designed the experiments: YZ and S-hS. Performed the experiments: X-jZ, YZ, JL, YG, and Y-hF. Analyzed the data: X-jZ and YZ. Wrote and revised the manuscript: X-jZ, J-sH, and Y-sZ. All authors read and approved the final manuscript.
